# Leaden Canula in the Treatment of Fistula in Ano

**Published:** 1842-05

**Authors:** 


### LEADEN CANULA IN THE TREATMENT OF FISTULA IN ANO.

Dr. J. W. Letton, of Lexington, Mo., has given us the history of fistula in ano, successfully treated by the introduction of a leaden tube. The purulent discharge from the bowels was profuse; and the cavity on the outside was several inches deep. At the suggestion of Dr. Ward, a leaden canula, three and a half inches long and two lines in diameter, was introduced and maintained in its position by a T bandage. The purulent discharge rapidly declined, granulations quickly began to fill up the bottom of the cavity, a frequent shortening of the tube became necessary, and in 10 days, it was finally withdrawn, the fistula not being more than half an inch deep. Cicatrization soon followed, and the cure was perfect and permanent.

